# Intense Resistance Exercise Promotes the Acute and Transient Nuclear Translocation of Small Ubiquitin-Related Modifier (SUMO)-1 in Human Myofibres

**DOI:** 10.3390/ijms17050646

**Published:** 2016-04-29

**Authors:** Sebastian Gehlert, Franz Josef Klinz, Lena Willkomm, Thorsten Schiffer, Frank Suhr, Wilhelm Bloch

**Affiliations:** 1 Institute of Cardiovascular Research and Sport Medicine, Department of Molecular and Cellular Sport Medicine, German Sport University Cologne, 50933 Cologne, Germany; williwictim@googlemail.com (L.W.); suhr@dshs-koeln.de (F.S.); w.bloch@dshs-koeln.de (W.B.); 2 Institute for Dental Research and Oral Musculoskeletal Biology, University of Cologne, 50935 Cologne, Germany; aka33@uni-koeln.de; 3 Outpatient Clinic for Sports Traumatology and Public health Consultation, German Sport University Cologne, 50933 Cologne, Germany; t.schiffer@dshs-koeln.de

**Keywords:** SUMO-1, resistance exercise, sumoylation, skeletal muscle adaptation, nuclear translocation, acute response

## Abstract

Protein sumoylation is a posttranslational modification triggered by cellular stress. Because general information concerning the role of small ubiquitin-related modifier (SUMO) proteins in adult skeletal muscle is sparse, we investigated whether SUMO-1 proteins will be subjected to time-dependent changes in their subcellular localization in sarcoplasmic and nuclear compartments of human type I and II skeletal muscle fibers in response to acute stimulation by resistance exercise (RE). Skeletal muscle biopsies were taken at baseline (PRE), 15, 30, 60, 240 min and 24 h post RE from 6 male subjects subjected to a single bout of one-legged knee extensions. SUMO-1 localization was determined via immunohistochemistry and confocal laser microscopy. At baseline SUMO-1 was localized in perinuclear regions of myonuclei. Within 15 and up to 60 min post exercise, nuclear SUMO-1 localization was significantly increased (*p* < 0.01), declining towards baseline levels within 240 min post exercise. Sarcoplasmic SUMO-1 localization was increased at 15 min post exercise in type I and up to 30 min post RE in type II myofibres. The changing localization of SUMO-1 proteins acutely after intense muscle contractions points to a role for SUMO proteins in the acute regulation of the skeletal muscle proteome after exercise.

## 1. Introduction

The small ubiquitin-related modifier protein family (SUMO) is essentially involved in the maintenance of the cellular proteome [1,2,3]. Similar to ubiquitination, SUMO proteins modify protein function and localization by reversible covalent binding to specific binding motifs on target proteins [3,4]. Sumoylation has been shown to be triggered in cells by osmotic and oxidative stress as well as acute hypoxia [5,6]. The function of sumoylated proteins depends on the type of target as well as its subcellular localization [7], but in contrast to ubiquitination, SUMO-conjugation prevents protein degradation and enhances protein stability [8,9,10]. One of the first reports on sumoylation demonstrated that RanGAP1 is modified by SUMO-1 [11], leading to its localization to the nuclear pore complex, where it is involved in the regulation of nucleo-cytoplasmic shuttling [12]. Sumoylated proteins and transcription factors are dominantly localized or translocated towards nuclei [13,14,15] as sumoylation acts e.g., by repressing [16] or enhancing transcriptional rates [17].

However, proteins which are typically localized in the cytosol or mitochondria, e.g., ribosomal proteins, glycolytic enzymes, and adenosine triphosphate (ATP) synthase subunits, are targets for sumoylation [18]. Interestingly, sumoylation has recently been reported to be involved in sarcoplasmic/endoplasmic reticulum calcium ATPase (SERCA) modification in failing heart muscle [19], providing a crucial role for calcium handling of contractile muscle tissue especially under conditions of increased stress. Additionally, a recent study showed that α-actin is sumoylated in rat skeletal muscle [20], providing an as yet undetermined role for modulation of contractility or protein stability.

Although a growing number of studies investigate sumoylation *in vitro* [13] or in animals [6], much less is known about SUMO regulation within adult human skeletal muscle. Moreover, in contrast to various aspects of protein signaling and gene expression in resistance exercised muscle tissue [8,9,10,21,22,23,24], basic information concerning the general response, time course and localization of SUMO proteins in response to exercise is missing.

Acute resistance exercise provides a mixture of mechanical [25], metabolic [26,27] and oxidative perturbations [28] within myofibres, acutely exerting the activation of signaling pathways [29], leading to increased transcription [30,31,32], protein degradation [22] and synthesis [33]. Because the regulation of skeletal muscle adaptation requires a complex network of protein translocation, degradation and maintenance, we hypothesized that SUMO proteins may also act as modulators in the early molecular response of loaded skeletal muscle, associated and reflected by the acute translocation of SUMO-proteins within skeletal muscle. The present study investigated the localization of SUMO-1 proteins in resistance-exercised human myofibres via immunohistological methods. Here we show that a single bout of high-intensity resistance exercise induces substantial nuclear translocation of SUMO-1 in skeletal muscle myofibres in an early time course after stimulation and describe a potential role of sumoylation in the acute response of skeletal muscle tissue towards exercise.

## 2. Results

### 2.1. Immunohistochemistry

**Figure 1:**
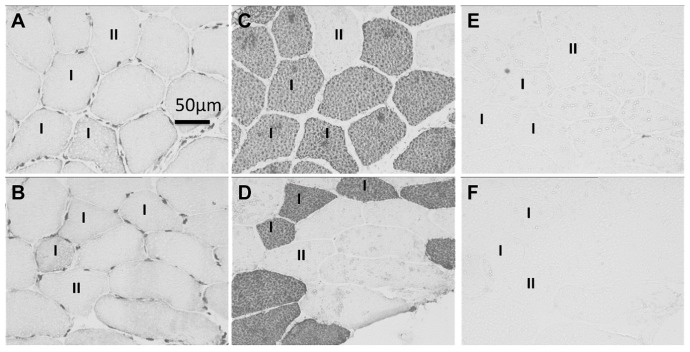
Small ubiquitin-related modifier (SUMO)-1 stained myofibre cross-sections in human skeletal muscle. (**A**,**B**) Skeletal muscle cross-section showing nuclear and sarcoplasmic staining of SUMO-1 antibodies in type I and II myofibres 15 and 30 min after resistance exercise, respectively; (**C**,**D**) Consecutive cross-sections stained for type I MyHc (A 4.840) showing type I (dark) and type II (bright) myofibres; (**E**,**F**) Consecutive cross-sections showing unstained negative controls of corresponding areas from (**A**,**B**).

Immunohistochemical staining of skeletal muscle cross-sections with SUMO-1 antibodies revealed specific SUMO-1 staining in sarcoplasmic and nuclear compartments of type I and II skeletal muscle fibers when compared to negative controls (Figure 1A,B specific SUMO-1 staining *vs.* MyHC stained consecutive cross-sections in C and D and consecutive cross-sections showing negative controls in E and F).

**Figure 2:**
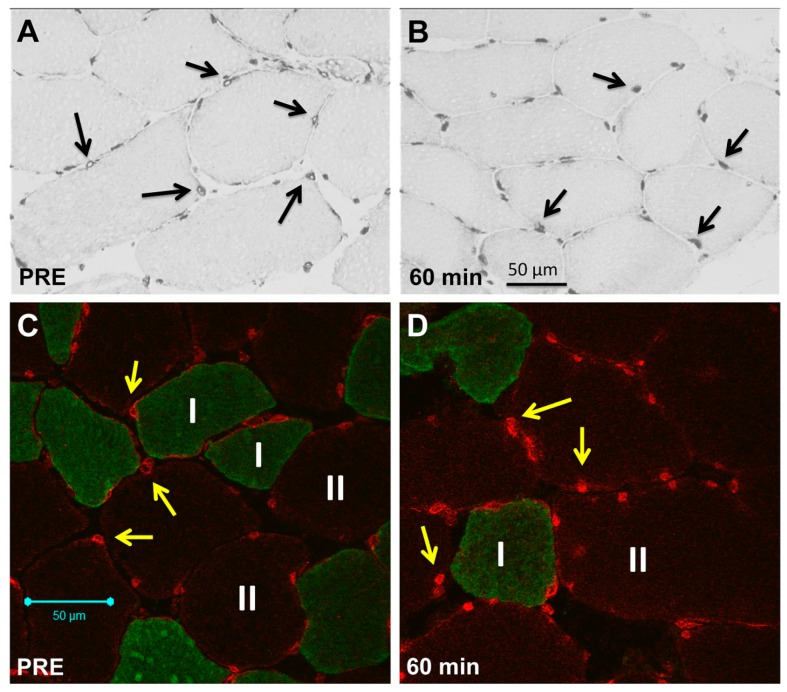
Immunohistochemical and immunofluorescence staining of SUMO-1 in skeletal muscle myofibres. (**A**) Diaminobenzidine (DAB)-stained consecutive cross-section showing nuclear and sarcoplasmic SUMO-1 staining in myofibres (200× magnification) under resting conditions (PRE) with lowered nuclear localization (black arrows indicating myonuclei with domiant perinuclear accumulation under baseline conditions); (**B**) DAB staining (200× magnification) of myofibres 60 min post resistance exercise (RE) showing increased nuclear accumulation of SUMO-1 (black arrows showing nuclei with increased intranuclear SUMO-1 localization); (**C**) Representative immunofluorescence staining of SUMO-1 in myofibre cross-sections under resting conditions (PRE) showing the preferential localization of SUMO-1 (Alexa 555, Red) at the nuclear envelope (yellow arrows) and in perinuclear regions of myofibres (MyHC I, Alexa 488, Green) (200× magnification); (**D**) Immunofluorescence staining (200× magnification) of myofibres 60 min post RE showing increased nuclear accumulation of SUMO-1 (Alexa 555, Red) and expanded SUMO-1 stained nuclear areas (yellow arrows).

Under resting conditions, SUMO-1 localization showed a predominant perinuclear localization and lower intranuclear abundance (Figure 2A,C), but showed a time-dependent increase in accumulated nuclear area in an early time course after resistance exercise (Figure 2B,D).

### 2.2. Sarcoplasmic Small Ubiquitin-Related Modifier (SUMO)-1-1 Localization

**Figure 3:**
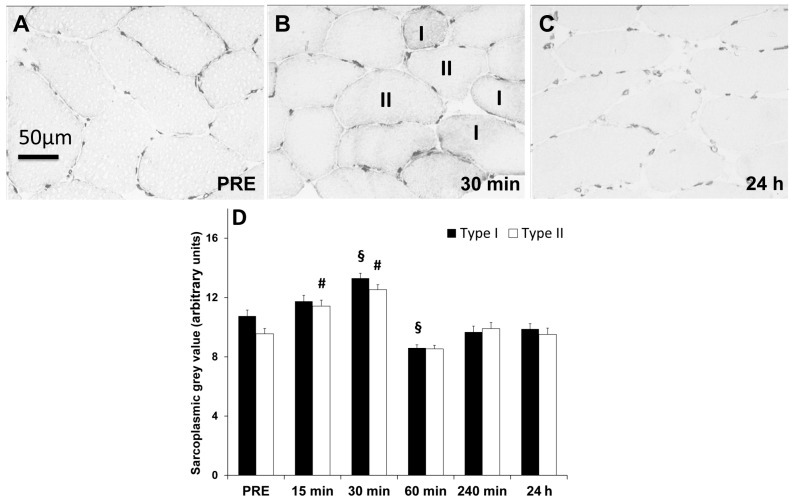
Changing sarcoplasmic SUMO-1 abundance within myofibres acutely and up to 24 h post resistance exercise. DAB-stained skeletal muscle cross-sections with changing sarcoplasmic SUMO-1 abundance after resistance exercise. (**A**) Skeletal muscle fibres displaying sarcoplasmic and nuclear SUMO-1 localization under resting conditions; (**B**) 30 min after cessation of RE with increased sarcoplasmic and nuclear density and (**C**) 24 h post RE displaying decreased sarcoplasmic and nuclear SUMO-1 localization; (**D**) Quantification of SUMO-1 abundance in sarcoplasmic compartments of type I and II myofibres over the time course of 24 h post RE. Values showing mean ± standard error of means (S.E.M.) arbitrary grey value units. Symbols: (§) time point significantly different (*p* < 0.01) compared to PRE in type I myofibres; (#) time point significantly different (*p* < 0.01) compared to PRE in type II myofibres; (horizontal bars) time point significantly different (*p* < 0.01) between type I and II myofibres.

**Figure 4:**
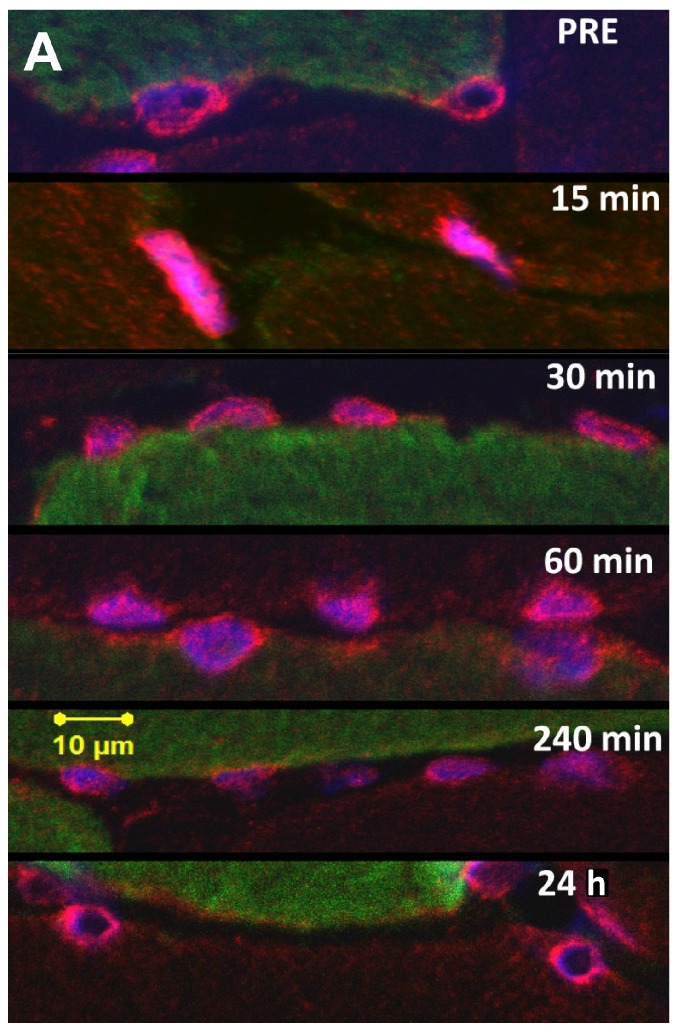

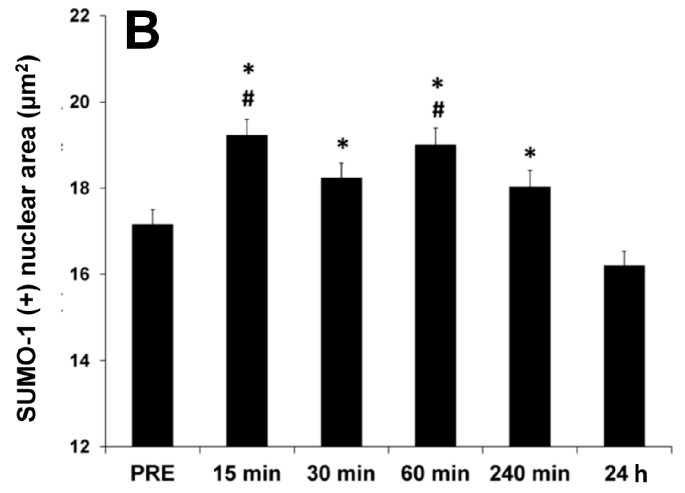
Representative immunofluorescence staining of SUMO-1 in myonuclei and quantification of SUMO-1 accumulated nuclear area. (**A**) Representative images of nuclear SUMO-1 localization within skeletal muscle myonuclei at baseline (PRE) as well as, 15, 30, 60, 240 min and 24 h post RE (630× magnification). SUMO-1 (Alexa 555, Red), myonuclei (Draq5, Blue) and MyHC I (Alexa 488, Green). SUMO-1 localization at PRE and 24 h post RE was preferentially located in perinuclear regions and closely to the nuclear envelope. Nuclear SUMO-1 localization increased from 15 min up to 60 min post resistance exercise but was reduced at 240 min and associated with a significant reduction of intranuclear SUMO-1 accumulation within 24 h; (**B**) Quantification of SUMO-1 stained nuclear area as determined in DAB-stained myofibres (compare Figure 2A,B) from PRE to 24 h post RE (Mean ± S.E.M). (#) significant different compared to PRE for (*p* < 0.01); (*) significant different compared to 24 h values (*p* < 0.01).

Within 30 min after RE, SUMO-1 density increased in sarcoplasm of both myofibre types (type I, *p* < 0.01, ῃ^2^ = 0.30; type II, *p* < 0.01, ῃ^2^ = 0.23) but decreased within 24 h post exercise towards baseline (*p* < 0.01) (Figure 3A–C and Figure 4A).

Type I myofibres offered at baseline (PRE) higher (*p* < 0.01) sarcoplasmic SUMO-1 localization than type II myofibres and tended to be generally higher in this fibre type after resistance exercise (Figure 1A,B and Figure 3B). However, relative increases tended to be higher in type II fibers (not significant). Sarcoplasmic SUMO-1 levels returned within 240 min and up to 24 h post RE towards baseline levels in both myofibre types (Figure 3C).

### 2.3. Nuclear SUMO-1 Localization

**Figure 5:**
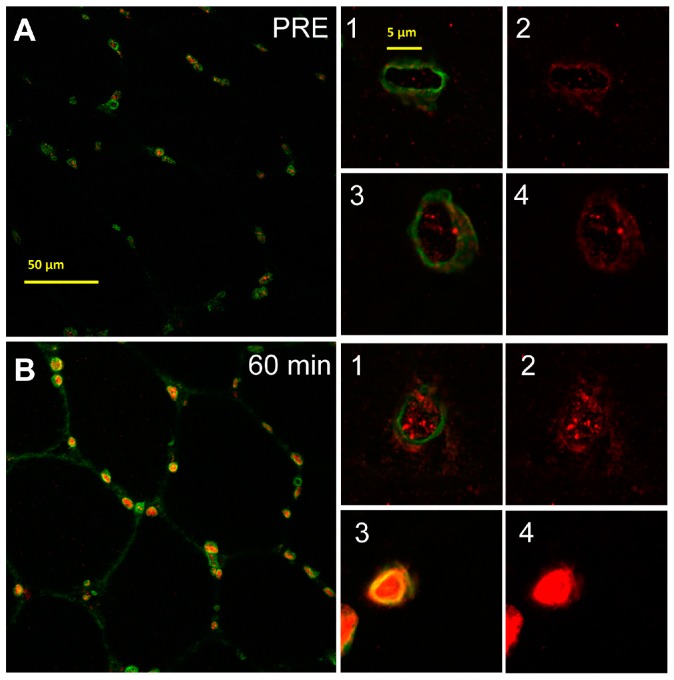

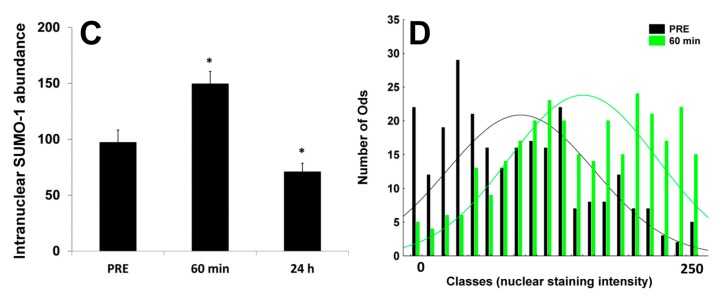
Representative immunofluorescence staining of SUMO-1 and lamin-A in myonuclei (SUMO-1 labelled with Alexa 555, Red, lamin-A labelled with Alexa 488, Green). Quantification of intranuclear SUMO-1 accumulation. (**A**) Representative images of nuclear membrane staining with lamin-A and intranuclear SUMO-1 localization within skeletal muscle myonuclei under resting conditions (PRE) and (Figure 5A, merge between lamin-A and SUMO-1, 400× magnification; Figure 5(A1–3) merge and Figure 5(A2–4) SUMO-1 signal from 1–3); (**B**) 60 min post RE. (Figure 5B, merge between lamin-A and SUMO-1, 400× magnification; Figure 5(B1–3) merge and Figure 5(B2–4) SUMO-1 signal from 1–3); (**C**) Quantification of intranuclear SUMO-1 localization within myonuclei at PRE, 60 min and 24 h post RE. (*) Significantly different from PRE values (*p* < 0.01); (**D**) Histogram showing the frequency of analyzed myonuclei with increasing intranuclear SUMO-1 abundance at 60 min post RE (bright green bars) when compared to PRE (black bars) from left to right. (0 = almost no nuclear SUMO-1 abundance, 250 = almost entire intranuclear abundance.)

Immunofluorescence staining showed a specific nuclear and perinuclear SUMO-1 localization in skeletal muscle nuclei already at baseline however with an increasing nuclear abundance of SUMO-1 in an early time-course after RE (Figure 2C,D, Figure 4A and Figure 5A,B). Quantification of SUMO-1 positive areas (independent of the fibre type, Figure 4B) revealed significantly expanding SUMO-1 accumulation from PRE (*p* < 0.01 and *p* < 0.05, ῃ^2^ = 0.069) up to 15 and 60 min after RE, respectively. From 240 min post RE nuclear SUMO-1 area decreased and at 24 h post there was a significant loss of accumulated nuclear SUMO-1 localization compared to 30 and 60 min values (*p* < 0.01 and *p* < 0.05).

To clearly distinguish between increased intranuclear SUMO-1 localization and accumulated SUMO-1 stained areas in perinuclear and near nuclear regions, we further conducted fluorescence staining of SUMO-1 in combination with lamin-A nuclear membrane staining (Figure 5(A1–4,B1–4)). Under resting conditions (Figure 5(A1–4)), SUMO-1 was predominantly localized in perinuclear regions and around the nuclear envelope showing a lowered intranuclear localization. At 60 min post RE (Figure 5(B1–4)) SUMO-1 was predominantly located within nuclei (Figure 5(B1–4)) or entirely filling the intranuclear area with additionally high abundance at the nuclear envelope. Quantification of intranuclear SUMO-1 abundance in skeletal muscle nuclei between the inner borders of the nuclear envelope (Figure 5C) revealed significantly increased intranuclear SUMO-1 levels from PRE to 60 min post RE (*p* < 0.01) and significantly reduced intranuclear levels at 24 h post RE (*p* < 0.01). Importantly, intranuclear SUMO-1 accumulation did not display a highly homogenous response within investigated nuclei but increased gradually and significantly in the vast majority of myofibres after RE. Figure 5D shows the number of myofibres with high intranuclear staining increase considerably at 60 min post RE compared to PRE.

## 3. Discussion

The response of SUMO proteins upon acute exercise in skeletal muscle is not described. Only one study observed increased SUMO-1 mRNA levels in response to 48 h of immobilization in human skeletal muscle [34] showing SUMO proteins to be involved in the regulation of atrophic skeletal muscle conditions. The investigation of SUMO-involving mechanisms in loaded skeletal muscle seems promising because SUMO-conjugation regulates a multitude of processes, all of which are stimulated by exercise, e.g., increased gene expression, protein signaling [32] and trafficking [35,36]. Although the determination of specifically-sumoylated targets was beyond the scope of this study, we determined, via an exploratory but purely descriptive approach, the time-dependent change of SUMO-1 localization in nuclear and sarcoplasmic compartments of human skeletal muscle fibers as acute phase reaction to intense skeletal muscle stimulation by RE.

In resting skeletal muscle we observed SUMO-1 to be predominantly localized in the perinuclear region and the nuclear envelope, while less SUMO was detected within the nucleus. Because the localization of SUMO-1 changed early after exercise in both sarcoplasmic and nuclear myofibre compartments, this points to a role for an early and extended protein sumoylation in skeletal muscle that can be triggered by exercise-induced stress. Amongst others, we have shown recently that acute increases in mechanical tension by RE induces increased myocellular signaling [21] and gene expression [22,37] within 15 min after cessation of RE [37,38,39,40,41], also accompanied by myofibrillar damage and upregulation of the protein degradation machinery chaperone-assisted selective autophagy CASA [22]. All of these processes are accompanied by a complex and overlapping network of post-transcriptional and post-translational modification of target proteins in which sumoylation may play a supporting role [7,14,15,42,43]. Thus, many nuclear and cytosolic proteins in skeletal muscle that are crucially involved in the regulation of hypoxia [44], energy metabolism [43], myofibre contractility [19], mitochondrial [45] or myocellular adaptation towards resistance exercise [46,47] are also known to be targeted by sumoylation *in vitro* [5,13,14,18] and *in vivo* [6,19]. Sustained myofibre contractions require the acute functional regulation of enzymes involved in energy metabolism, ATPsynthase subunits, calcium channels and contractile filaments. These proteins are highly abundant in the sarcoplasm and have been shown to be sumoylated in contractile tissues [6,18,20]. Hence, augmented SUMO-conjugation of these proteins might have contributed to the observed increase in SUMO-1 localization in sarcoplasm of human type I and II myofibres.

In this context sumoylation of α-actin [20] might have contributed to the observed sarcoplasmic SUMO-1-staining. It might be hypothesized that especially under conditions of contractile stress, sumoylation of actin filaments could act by transiently preserving for ubiquitin-dependent degradation. Interestingly, type I fibers showed a partly higher SUMO-1 staining pattern than type II fibers and might be explained by the generally higher protein content of type I fibers due to increased mitochondrial density. Both myofibre types showed a similar increase in sarcoplasmic SUMO-1 localization after RE and exercise-dependent differences between fiber types in the regulation of sarcoplasmic SUMO-1 localization remain unclear.

The early response of skeletal muscle myofibres towards resistance exercise-stimulation affected especially also the myonuclear compartment. The modulation of gene transcription requires the nuclear translocation or nuclear removal of targets via nucleo-cytoplasmic shuttling. Both processes have been described as being controlled in part by SUMO [9,12,48]. Cytoplasmic-nuclear shuttling includes myocyte-specific enhancer factor (MEF)-2, peroxisome proliferator-activated receptor gamma coactivator (PGC)-1-α, p38 and c-Jun, [13,14,15,49] representing designated targets for sumoylation and play important roles in the adaptation of mitochondria and skeletal muscle towards exercise [50,51]. Activity-induced myofibre shifting and mitochondrial biogenesis e.g., is increased after cessation of acute exercise and require the aforementioned nuclear translocation and regulation of transcription factors. It has been proposed that sumoylation of type II histone deacetylase proteins (HDAC2) might lead to MEF2 sumoylation and inactivation within nuclei, counteracting exercise-induced slow shifting of myofibres [52].

Nuclear translocation of transcription factors that are crucial for skeletal muscle adaptability occurs within the first hours after exercise [35,36,53], a time course in which we observed a significant increase of SUMO-1 localization in myonuclei. Therefore it might be hypothesized that the observed change in SUMO-1 localization and density will have involved sumoylation and subsequent translocation of some of the aforementioned targets. Future studies are required to describe these events in exercising muscle more specific, with regard to the identification of sumoylated targets, and importantly, describing the cellular and physiological consequences of sumoylation for myocellular adaptation. Although explorative in nature, the present investigation is the first to describe the time course and localization of SUMO-1 in compartments of human myofibres in response to acute resistance exercise. Our results emphasize a role for SUMO proteins in early cellular responses within the first hours after skeletal muscle stimulation with potential implication for maintenance and regulation of exercise-induced adaptation in skeletal muscle.

## 4. Materials and Methods

### 4.1. Study Design

**Figure 6:**
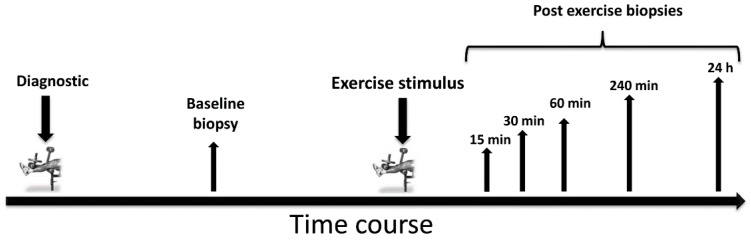
Baseline strength diagnostics and skeletal muscle biopsies were collected 7–10 days prior to the exercise intervention. After application of the main resistance exercise session, several skeletal muscle biopsies were collected from the exercised leg within 24 h.

Figure 6 displays the schematic overview of the study design.

### 4.2. Subjects

Six healthy male subjects (age: 23 ± 4 years, height: 180 ± 89 cm, and weight: 79 ± 10 kg) participated in our study. Subjects were informed orally and in writing of the study’s purpose and the possible risks involved before providing informed consent. Prior participation all subjects provided written and verbal informed consent to take part in the intervention. Written informed consent document were collected and stored in the office of the head of the institute. The study (BISPIIA1 070103/09-10) was approved by the Ethics Committee of the German Sport University Cologne in compliance with the Declaration of Helsinki and approved all documents for subject information and informed consent prior handling out to the subjects.

### 4.3. Standardization of Diet and Activity before Exercise

Subjects were instructed to refrain from RE 14 days prior to this study and from any physical activity 48 h prior to baseline biopsies, exercise testing and the main RE stimulus. The day prior the resting biopsy as well as the main exercise stimulus a standardized protein-energy drink (Fresubin^®^ protein energy drink, Fresenius Kabi Deutschland GmbH, Bad Homburg, Germany; containing 20 g protein, 24.8 g carbohydrate 13.4 g fat, providing 1260 kJ) was provided to the subjects at 22:00 before they fasted overnight. The following morning, one hour prior to the resting biopsy and the main RE intervention, subjects were advised to drink a second energy drink in order to carry out the exercise in the fed state. Subjects were allowed to drink water *ad libitum* during all exercise interventions.

### 4.4. Experimental Trials

On the day of the experimental trial, subjects arrived at the laboratory at 07:45 AM. Subjects were instructed to refrain from vigorous physical activity for two days prior to baseline biopsies and the RE intervention. Prior to RE, a standardized warm-up program on a cycle ergometer was applied (5 min cycling with 1 W/kg bodyweight). After a 3 min resting phase, the RE protocol was performed containing one single set with 20 concentric and eccentric knee extensions with maximum voluntary force. The angular velocity was set to 40°/seconds (s) during the eccentric and concentric movement phases of knee extensions, which provided in sum 70 s of time under tension. Subjects were verbally encouraged to perform all repetitions with maximum voluntary force until exhaustion. After local anesthesia several muscle biopsies were collected from the vastus lateralis muscle of the exercised leg.

### 4.5. Skeletal Muscle Biopsies

Baseline biopsies of the vastus lateralis muscle of the exercising leg were taken at rest, ten days prior to the exercise intervention using the percutaneous needle biopsy technique. On the day of the experimental trial five biopsies were collected, at 15, 30, 60, 240 min and 24 h after cessation of the resistance exercise protocol. 15, 30 and 60 min biopsies were taken from the same incision 2 cm distal of the resting biopsy. The angle of the biopsy needle was altered during every biopsy with the needle pointing straight (15 min), inward (30 min) and outward (60 min) to collect samples from unaffected regions. The 240 min biopsy sample was collected 2 cm proximal of the resting biopsy incision and the 24 h biopsy was collected distal from 15, 30 and 60 min incision. An outside routed scale allowed standardization of the biopsy depth for each subject. Muscle biopsies were obtained from the middle region of the vastus lateralis muscle between the spina iliaca anterior superior and the lateral part of the patella, 3 cm below entry through the fascia.

### 4.6. Tissue Processing and Staining

Muscle samples were freed from blood and non-muscle material, embedded in Tissue-Tek (Sakura Finetek, Zoeterwoude, The Netherlands), frozen in liquid nitrogen-cooled isopentane and stored in liquid nitrogen until further processing. For immunohistochemical staining procedures, 7 µm cross-sections of all biopsy time points were mounted in double on Polysine^®^ slides (VWR International GmbH, Darmstadt, Germany), carefully aligned for cross-sectional analysis, air-dried and stored at −80 °C. Immunohistochemical procedures were conducted according to previous works [21,54]. Skeletal muscle cross-sections were incubated overnight at 4 °C with specific primary antibodies recognizing SUMO-1 (#4930; Cell Signaling, Beverly, MA, USA), nuclear envelope marker lamin-A (ab26300, Abcam, Cambridge, UK) and A 4.951, which is raised against adult human slow myosin heavy chain (MyHC1) (Developmental Studies Hybridoma Bank, Iowa City, IA, USA). Antibodies were diluted 1:150 (SUMO-1), 1:200 (lamin-A) or 1:200 (A4.951) in TBS containing 1% bovine serum albumin (BSA). Muscle cross-sections from all biopsy time points of each subject were stained within a single batch to minimize variability in staining efficiency. Double-mounted, consecutive serial sections on the same slide were stained simultaneously for slow MyHC1 and SUMO-1.

### 4.7. Analysis of Nuclear and Sarcoplasmic SUMO-1 Localization

Stained cross-sections were examined with a Zeiss Axiophot 200 light microscope coupled to a Sony 3CCD Color Video Camera (AVT Horn, Aalen, Germany). Digitally captured images (8 bit-grayscale) (200× magnification) with 8 fields of view per muscle cross-section (9 ± 3 fibers per field of view) were analyzed. The specific staining intensity for SUMO-1 stained type I and II myofibres was quantified by selection of the sarcoplasmic part of the investigated myofibres. Optical densitometry was carried out with the software ImageJ^®^ (National Institutes of Health, Bethesda, MD, USA). Sarcoplasmic SUMO-1 density in each myofibre was expressed as mean staining intensity in 8-bit greyscale pictures with 256 grades of resolution (0 = entirely black, 256 = entirely white). Data show the netto staining intensity shown as arbitrary data, determining the difference between equalized background light transmission (adjusted equally for each cross-section to an equal value of 220) and the specific light transmission within each myofiber. In sum, 614 type I and 612 type II myofibres were analyzed with a mean number of 102 ± 4 type I and 102 ± 3 type II myofibres for each time point and a similar number of fibers per subject. Mean values and standard deviation were calculated by the mean value for all fibers within that time-point. Quantification of nuclear SUMO-1 area was carried out in DAB-stained cross-sections by automatic analysis of SUMO-1 stained nuclear regions via the particle analysis option within ImageJ^®^ (National Institute of Health). 16-Bit pictures (200-fold magnification) of all subjects and time points were analyzed with identical grey-value threshold for determining pixel as SUMO-1 positively stained. In prior analysis, SUMO-1 stained areas were determined as exact measures of specific nuclear SUMO-1 staining but excluding non-specific and small sarcoplasmic areas. The determined values constituted the area (μm^2^) of specific SUMO-1 stained pixels within or around nuclear compartments and within the applied staining-threshold for each picture, time point and subject. 819 ± 141 SUMO-1 stained nuclear areas were automatically counted for each time point in all subjects and analyzed with STATISTICA 7 analysis software (Statsoft, Tulsa, OK, USA).

### 4.8. Immunofluorescence Analysis

Tissue preparation and fluorescence staining was conducted as previously described [21]. Alexa488 goat anti-mouse (Invitrogen, Karlsruhe, Germany; dilution 1:500) and Alexa555 goat anti-rabbit (Invitrogen, Karlsruhe, Germany; dilution 1:500) secondary antibodies were used to determine type I myofibres, lamin-A and SUMO-1 respectively. Pictures were taken by a Zeiss confocal laser scanning microscope equipped with Plan-Neofluar 40× and 63×/1.3 Oil DIC objectives (LSM 510Meta, Zeiss, Jena, Germany). Alexa488 was excited by an Argon laser using the filter set BP505-530, Alexa555 by a Neon laser using the filter set BP565-615. Intranuclear SUMO-1localization was conducted by determining the fluorescence staining intensity of SUMO-1 (244 ± 54 nuclei per time-point of subjects) doubly-labeled for SUMO-1 (Alexa 555) and lamin-A (Alexa 488) in pictures with 40× fold magnification. The quantification of intranuclear SUMO-1 signal was conducted via ImageJ^®^ by using the line scan function exclusively within the inner borders of lamin-A stained nuclear envelope of nuclei.

### 4.9. Statistics

Data are expressed as means ± standard error of means (S.E.M.). Multifactorial analysis of variances (ANOVA) for repeated measures with the factors “time” and “fiber type” was applied to determine differences in the density of SUMO-1 in the sarcoplasmic compartment of muscle fiber types over time. One-way ANOVA with the factor time was applied to determine differences in the area of SUMO-1 stained nuclear compartments of myofibres. Bonferroni post hoc tests were used to locate the differences. Significance was assumed at *p* < 0.05. Results of the ANOVA include the effect size (partial ῃ^2^) which was corrected by Greenhouse-Geisser correction if sphericity was violated.
